# Engagement of the private pharmaceutical sector for TB control: rhetoric or reality?

**DOI:** 10.1186/s40545-016-0093-3

**Published:** 2017-01-18

**Authors:** Niranjan Konduri, Emily Delmotte, Edmund Rutta

**Affiliations:** Systems for Improved Access to Pharmaceuticals and Services (SIAPS) Program, Management Sciences for Health, 4301 N. Fairfax Dr. Suite 400, Arlington, VA 22203 USA

**Keywords:** Public-private mix, Tuberculosis, Pharmacists, Retail drug outlets, Private sector, Pharmacy associations

## Abstract

**Background:**

Private-sector retail drug outlets are often the first point of contact for common health ailments, including tuberculosis (TB). Systematic reviews on public-private mix (PPM) interventions for TB did not perform in-depth reviews specifically on engaging retail drug outlets and related stakeholders in the pharmaceutical sector. Our objective was to better understand the extent to which the World Health Organization’s (WHO) recommendation on engaging retail drug outlets has been translated into programmatic policy, strategy, and intervention in low- and middle-income countries.

**Methods:**

The study included a content analysis of global-level documents from WHO and the Stop TB Partnership in five phases. A country-level content analysis from four data sources was performed. Global-level findings were tabulated based on key messages related to engaging retail drug outlets. Country-level findings were analyzed based on four factors and tabulated. National strategic plans for TB control from 14 countries with varying TB burdens and a strong private sector were reviewed.

**Results:**

33 global-level documents and 77 full-text articles and Union World Lung Health conference abstracts were included for review. Based on experience of engaging retail drug outlets that has emerged since the mid-2000s, in 2011 WHO and the International Pharmaceutical Federation released a joint statement on promoting the engagement of national pharmacy associations in partnership with national TB programs. Only two of 14 countries’ national strategic plans had explicit statements on the need to engage their national pharmacy professional association. The success rate of referrals from retail drug outlets who visited an approved health facility for TB screening ranged from 48% in Vietnam to 86% in Myanmar. Coverage of retail drug outlets ranged from less than 5 to 9% of the universe of retail drug outlets.

**Conclusions:**

For WHO’s End TB Strategy to be successful, scaling up retail drug outlets to increase national coverage, at least in countries with a thriving private sector, will be instrumental in accelerating the early detection and referral of the 3 million missing TB cases. The proposed PPM pharmacy model is applicable not only for TB control but also to tackle the antimicrobial resistance crisis in these countries.

**Electronic supplementary material:**

The online version of this article (doi:10.1186/s40545-016-0093-3) contains supplementary material, which is available to authorized users.

## Background

There is no shortage of evidence that both regulated and unregulated private-sector retail drug outlets, also known as pharmacies, chemists, drug shops, drug sellers, drug vendors, or informal drug sellers, are often the preferred first point of contact for common health ailments due to their inexpensive services, ease of access, and lack of waiting times compared to public health facilities [1, 2],. There is substantial evidence regarding the health-seeking behavior of consumers, caregivers, and patients [3] related to childhood illnesses, malaria [4], sexually transmitted diseases [5], cough-like symptoms [6], prolonged cough [7], and tuberculosis (TB) [8, 9],. Systematic reviews on the role of private-sector retail drug outlets in the provision of health care [10] and their quality of services [11] and regulatory aspects [12] have examined this body of knowledge.

While 43 million lives were saved by TB treatment and care between 2000 and 2014, TB remains a scourge worldwide, and 9.6 million people contracted the disease in 2014 [13]. To end the TB epidemic by 2035, the World Health Organization’s (WHO) End TB Strategy emphasizes tapping the full benefits of health policies and systems by engaging a much wider set of collaborators across government, communities, and the private sector. In 2013, WHO reported that 3 million people fail to get a quality-assured TB diagnosis each year and often seek care from multiple private-sector providers in their search for TB treatment. Private-sector retail drug outlets are a key ally in ensuring that patients are referred to appropriate and high-quality treatment providers in their neighborhood. WHO’s first guideline on public-private mix (PPM) approaches for tuberculosis clearly spells out the range of interventions specifically needed for non-physicians, such as private-sector retail drug outlets, pharmacists, and traditional healers [14]. However, the extent to which this specific recommendation has been translated into programmatic policy, strategy, and intervention in low- and middle-income countries is unclear.

A WHO review of PPM interventions found that TB detection increased between 10 and 36% with the successful treatment of 90% of new smear-positive pulmonary TB cases [15]. One systematic assessment examined the concept and practice of PPM and concluded that national TB programs (NTPs) need guidelines to make decisions on which type of provider to engage to meet the Stop TB Partnership’s global objectives [16]. A recent systematic review evaluated the performance of PPM programs against six health system themes [17]. Given the broader PPM focus, these studies did not perform in-depth reviews specifically on engaging private-sector retail drug outlets in TB control. The latter represent a particular challenge for PPM interventions because they exist in large numbers and are often staffed by low-skilled employees whose clients are looking for a quick remedy. Meanwhile, the PPM schemes ask the retail drug outlets to focus on referrals that bring no inherent benefit to the drug seller and sometimes resistance from the client.

To better understand the evolution of the recommendations concerning the engagement of private-sector retail drug outlets in TB control, we performed a content analysis of documentation from global agencies. We also reviewed and examined whether retail drug outlet engagement in TB control is merely rhetoric reflected in global guidelines and practiced in a handful of settings or reality reflected in country policy and practice. We examined whether NTPs, at least in countries with a large private sector, have embraced retail drug outlets and implemented interventions to engage them as part of their PPM strategy.

## Methods

### Global-level content analysis

We conducted the search in five phases. First, we reviewed all resources, tools, and publications available on WHO’s PPM web page. We also reviewed relevant documents from WHO’s Global TB program and Stop TB Partnership web pages. We performed a content analysis of all relevant documents for recommendations and manually scanned all documents to verify statements related to the purpose of this review. Second, we reviewed the publicly available WHO and Stop TB Partnership PPM subgroup meeting notes, or the agendas in the absence of meeting notes, from 2002 to 2015. We applied the search terms “pharmacy” and “pharmacist.” Third, we reviewed WHO’s annual global TB reports from 2000 to 2015 for the search terms “PPM”, “public-private partnerships”, “pharmacy”, “pharmacist”, “chemist”, and “private sector.” Fourth, we reviewed annual reports, strategic plans, and key Stop TB Partnership documents sourced from WHO’s website from 2003 to 2015. Fifth, we reviewed 15 meeting reports and recommendations from WHO’s Strategic and Technical Advisory Group for Tuberculosis from 2001 to 2015. In all instances, we hand searched additional documents mentioned or described in these documents and added them for review as appropriate.

### Country-level content analysis

We searched PubMed and Google Scholar using free words at the time of this review (see Additional file 1 for search terms). For PubMed, an advanced search was used to find additional articles with the terms included in the title or abstract. We hand searched the bibliographies of relevant articles and contacted study authors for additional clarification. All articles were screened by one reviewer (ED) and verified by another (NK). Article inclusion criteria were those articles related to retail drug outlets and could be a commentary, an assessment of the situation, or an intervention. All other articles that had other PPM components or described such things as health-seeking behavior or diagnostic delays were excluded. Because we anticipated that there would be very few studies or articles that focused on retail drug outlets and TB control or included them as part of a broader PPM intervention, we expanded our search through three sources. First, we searched the abstract books of the Union World Conference on Lung Health published by the *International Journal of Tuberculosis and Lung Disease* from 2004 to 2014. We searched abstracts for the following terms: “pharmacy”, “pharmacist”, “chemist”, “shops”, and “seller”. Second, we collected available national TB strategic plans or action plans specifically from countries with high TB burden and focused on 14 such countries where the private expenditure for health was at least 45% or more of the total health expenditure and assessed country policy on PPM in relation to private pharmacy engagement [18]. Third, we retrieved the estimated number of licensed and unlicensed retail drug outlets relative to the country population against the TB burden for 13 of 14 countries (excluding India) to permit comparisons. The latter analysis was performed to assess public health implications of the relative level of scaling up PPM interventions involving retail drug outlets.

In this review, we consistently use the term “retail drug outlets” to reflect the various terms used in the literature, including drug seller, drug shop, medicine store, private pharmacy, chemist, informal drug seller, patent drug vendor, patent medicine vendor, and accredited drug dispensing outlet. However, during the multi-stage search process, we used these varying terms to identify relevant documents. From a regulatory standpoint, in the majority of the countries, outlets are generally classified into two categories. Category I includes those outlets that are legally allowed to sell only non-prescription medicine, also known as over-the-counter drugs, while Category II includes those outlets that are legally allowed to sell prescription medicines. In practice, there may often be no distinction in terms of what is being sold, but many other legal requirements are maintained.

## Results

### Global documentation: WHO and the Stop TB Partnership

A total of 33 global-level documents were reviewed. The content analysis of 16 key documents from the WHO and Stop TB Partnership websites is presented in Table 1. Pharmacists were first included as part of the formal definition of private providers in WHO’s 2001 emerging policy framework to involve private practitioners [19]. A summary of the notes and presentations from eight Stop TB Partnership PPM meetings is shown in Table 2. Aspects of private pharmacy engagement were addressed in 2002, 2006, and from 2010 to 2014 in 11 Stop TB PPM subgroup meetings. The PPM subgroup dedicated significant time during the 2011 subgroup meeting to discussing and sharing experiences on anti-TB drugs and the private sector and engaging pharmacists. This meeting resulted in a concrete recommendation to the Directly Observed Treatment Short Course (DOTS) Expansion Working Group and the Stop TB Coordinating Board to disseminate widely the WHO/International Pharmaceutical Federation (FIP) joint statement on promoting the engagement of pharmacy associations and drug regulatory bodies in national partnerships to stop TB.

**Table 1:** Key Documents from the WHO and Stop TB Partnership Websites

Key documents	Key messages related to engaging private-sector retail drug outlets	Gaps
TB patients and private providers in India (1997) [44]	Exclude anti-TB drugs from private channels. Prescriber-oriented education in private drug-distribution channels. Delegation of TB control responsibilities to non-governmental organizations. Public-private collaboration for the delivery of documented TB cures.	No recommendation of engaging “drug retailers” despite documenting evidence of their TB drug dispensing practices.
Global Plan to Stop TB (2001–2005) [45]	DOTS strategy implementation specified for private practitioners, non-governmental organizations, hospitals, clinics, prisons, industry, and military.	No explicit mention of engaging private pharmacies.
Legislation and Regulation for TB Control (2001) [46]	Create an effective partnership with private-sector physicians to implement national guidelines on TB control. Envisage the regulation of a drug supply for TB exclusively through the public health system.	No mention of engaging private pharmacies.
Emerging policy framework for involving private practitioners (2001) [19]	First WHO document to include “private pharmacists” as part of the formal definition of private providers to be engaged in TB control. Global assessment in 23 countries focused on private physicians. Captured evidence on patient health-seeking behavior in pharmacies and unrestricted availability of anti-TB drugs.	Options for engagement prioritized only for physicians. Restriction on TB drug availability in the private sector specified without engagement of wholesalers and private pharmacies.
Improving TB Drug Management. Accelerating DOTS Expansion (2002) [47]	In the context of analyzing TB drug management practices and to inform decision-making, recommendations were made to monitor private pharmacies or private clinics if they are an important source of anti-TB drugs.	None
Expanded DOTS Framework (2002) [48]	Involve private-sector health providers for case detection and DOTS implementation.	No specification of private pharmacies as part of the private sector.
Expanding DOTS in a changing health system (2003) [49]	Considerations on how best to ensure standardized, high-quality, affordable drugs through all providers, including private pharmacies, will be necessary.	Engaging private pharmacies to ensure an uninterrupted supply of high-quality drugs was briefly considered in the context of the role of private providers. There was no mention of engaging private pharmacies from the perspective of patient case detection and referral.
PPM DOTS Practical Tool (2003) [50]	“Pharmacists” was mentioned several times throughout the document, including considerations on how to engage them. A sample referral form for non-physicians was included to encourage adaptation and use depending on the local context.	None
PPM Guidelines (2006) [51]	The guideline clearly lists the importance of engaging pharmacists, drug shops and non-physicians so that the poor and vulnerable can receive appropriate care and referrals. Interventions include identifying persons suspected of having TB, collecting sputum samples, making referrals, notifying/recording cases, and supervising treatment. Pharmacy associations were listed among various PPM stakeholders for engagement at the national level.	None
DOTS Expansion Working Group Strategic Plan (2006) [52]	The term “PPM DOTS” has evolved to represent a comprehensive approach to involve all relevant health care providers in DOTS. PPM-DOTS targets a wide range of audiences as well as private health care providers not yet sufficiently linked to NTPs. Private pharmacies were included among a variety of private providers.	None
Second Global Plan to Stop TB (2006) [53]	Promotes the wider and more strategic use of existing strategies for TB control with an explicit mention of engaging “private pharmacies” and the “informal health sector” for introducing or scaling up PPM-DOTS.	None
9th WHO STAG-TB Meeting (2009) [54]	Special session on policy change for improved quality and rational use of anti-TB drugs. Recommended to schedule anti-TB drugs as restricted with special reporting requirements for pharmacies and prescribers. WHO must develop approaches to engage pharmaceutical companies, professional associations, and pharmacies to curb unethical practices and promote rational use of anti-TB drugs.	None
PPM Scale up (2010) [55]	Non-physicians and private pharmacies were included as part of a PPM task-mix strategy. Pharmacists may be able to identify persons with TB-like symptoms, collect sputum samples, refer suspects, notify or record cases, and supervise treatment.	None
Third Global Plan to Stop TB (2011) [56]	There is good evidence that PPM approaches can increase the percentage of people who are diagnosed and receive high-quality treatment by between one-quarter and one-third, with health care providers, such as pharmacists, traditional healers, and private practitioners, often serving as the first point of contact for people with TB symptoms.	None
Role of pharmacists in TB care and control (2011) [57]	The WHO/FIP joint statement recommended engaging pharmacists and national pharmacy associations in TB control.	None
Engaging all providers for drug-resistant TB (DR-TB) (2015) [58]	Non-physicians, such as private pharmacists, are currently engaging in PPM for TB care and control. They can be similarly engaged in patient-centered care for DR-TB, such as by providing DOTS and identifying and reporting side-effects of second-line drugs. Pharmacists can also provide education to family members on infection control and strategies to prevent and manage stigma.	None

**Table 2:** Stop TB PPM Subgroup Meetings

Year	Aspects related to private-sector retail drug outlets
2002	Involvement of pharmacies was listed as an innovative approach. Incentives for pharmacy involvement in referrals and treatment.
2006	Pilot experience on engaging private pharmacies in Cambodia and drug vendors in Vietnam was mentioned.
2008	WHO Activities in the Americas Region: The experience of NTPs in engaging private pharmacies and traditional medicine is scarce. Mexico has an agreement with the national pharmacy association. Constraints in the Americas include limited knowledge of the role and coverage of the private sector, including private pharmacies.
	Donor perspective: USAID supported the following activities Cambodia: pharmacy staff and traditional healer training and referral systems. Ethiopia: training and referral systems for private pharmacies.
2010	Cambodia reported progress on engaging private doctors and pharmacists: 12,577 suspects were referred, 6,403 were evaluated, and 1,418 TB cases were identified (2005–2008). An analysis of patient health-seeking behavior helped to design the intervention. Ghana reported progress on working with regulatory authorities to restrict access to anti-TB drugs and to require private pharmacies to refer all persons suspected of having TB to the NTP.
2011	The terms “pharmacy” and “pharmacist” were mentioned 19 times in the meeting report and discussed frequently, as reflected in numerous presentations. The PPM secretariat was recommended to support the documentation and dissemination of innovative approaches, such as engaging pharmacists in TB care and control. NTPs and Ministries of Health were recommended to work with national pharmacy associations to tap the role of pharmacists in early case detection and improving TB treatment and care.
2012	One of the expected outcomes of the subgroup meeting was to produce practical tools on social franchising for and engaging pharmacies in TB care.
2013	Reported progress made on designing guidance and tools to engage private pharmacies.
2014	The meeting provided recommendations to address the knowledge gap on income sources and amounts for chemists to inform the types of incentives that might work. PPM programs must enforce regulation for the rational use of anti-TB drugs and accreditation systems for collaborating providers.

A summary of related information from WHO’s nine annual Global TB reports is shown in Table 3. The momentum on overall PPM approaches gained traction between 2005 and 2010, and emerging results were shown in the 2011 and 2012 reports. No annual reports from the Stop TB Partnership, including those from TB REACH grants, had any information or statements that were significantly related to the purpose of this review.

**Table 3:** WHO Annual Global TB Reports

Year	Private-sector pharmacy aspects
2005	Cambodia: Based on the findings of a 2002 study on the prevalence of health care-seeking behavior in the private-sector, Cambodia launched a pilot project to engage private practitioners and pharmacies. Kenya: Diagnostic and treatment services projects to engage all providers, including pharmacies, are ongoing.
2007	Cambodia: Planned activities include mapping the locations of private pharmacies and recording the training of non-NTP staff. The Philippines: Achievements include initiating operational research projects in PPM, including collaboration with pharmacies. South Africa: Achievements include engaging pharmacists, private-sector general medical practitioners, traditional health practitioners, community care givers, and community-based organizations in the referral and support of TB patients.
2008	Afghanistan: Achievements include conducting a study on the role of private pharmacies in the treatment of TB in the central region of Afghanistan. Planned activities include developing training modules for private practitioners and private pharmacies to engage all care providers. Kenya: Achievements include sensitizing pharmacists and additional private practitioners on TB to encourage the referral of TB suspects for diagnosis.
2010	Countries have prioritized different types of care providers, including pharmacies in Cambodia, private hospitals in Nigeria, public hospitals in China and Indonesia, social security organizations in Mexico, and prison services in Kazakhstan.
2011	In 20 countries for which data were available, PPM contributed approximately 20 to 40% of all notifications in 2010 in the geographical areas in which PPM was implemented *(no specific data for pharmacies).* The role of pharmacists in TB care and control was discussed, including a box summary outlining the WHO/FIP joint statement.
2012	Intensified efforts by NTPs to engage the full range of care providers using PPM initiatives are also important; in most of the 21 countries that provided data, 10 to 40% of national notifications were from non-NTP care providers (*no specific data for pharmacies).*
2013	No specific information pertaining to private-sector pharmacy engagement.
2014	No specific information pertaining to private-sector pharmacy engagement.
2015	In India, patients receive e-vouchers for standardized medications to be redeemed at no charge from private chemists.

### Country-level findings

PubMed identified 32 unique articles for the search terms that were used, and seven articles that had a retail drug outlet component were included for review. The Google Scholar search identified 62 unique articles related to TB in the private sector, and five relevant articles were included for review. Among the Union World Conference on Lung Health abstracts between 2004 and 2014, we found 65 relevant abstracts for review. Including the 12 full-text articles sourced from PubMed and Google Scholar and 65 Union abstracts, a total of 77 relevant articles and abstracts representing 18 countries were analyzed based on four factors and tabulated (Additional file 2). Of the 18 represented countries, 11 were in Asia, five in Africa, and two in Latin America. India (17) had the most articles and abstracts included in this review, followed by Cambodia (9). Among the 77 articles and abstracts, 52 were interventions involving retail drug outlets, and 24 were assessments concerning any aspect of retail drug outlets, such as sales of anti-TB drugs and knowledge of providers. Only one abstract described a regulatory component concerning the restriction of anti-TB drugs involving key stakeholders, including the national medicines regulatory authority.

Only 15 of the 52 intervention-related articles and abstracts reviewed explicitly documented the specific number or percentage of referrals of presumptive TB cases from retail drug outlets or resulting smear-positive TB cases (Table 4). All other articles and abstracts had grouped numbers or percentages of referrals among all private providers in their overarching PPM interventions, making it difficult to obtain data on the contribution of referrals specifically among retail drug outlets. Between 2003 and 2014, there was no substantial progression or variation in the number of referrals regardless of the country setting. The success rate of referred patients who visited an approved health facility for TB screening varied from 48% in Vietnam in 2003 to 86% in Myanmar in 2014.

**Table 4:** Number of Referrals or Smear-positive Cases from Retail Drug Outlets over Time

Country	Year	Number of retail drug outlets	Number of referrals	% screened among referrals	Smear-positive cases
Vietnam	2003	150	310	48% (149)	10
Bolivia	2005	70	41	27% (11)	3
Philippines	2005	no data	2,334	no data	no data
Philippines	2005	no data	1,550	37% (575)	83
Cambodia	2008	683	4,230	79% (3,356)	1,769
India (Tamil Nadu)	2010	402	101	no data	no data
Philippines	2011	119	942	11% (99)	14
India (2 cities)	2012	80	23	No data	8
Burkina Faso	2013	131	821	44% (361)	17
India (Andhra Pradesh)	2013	60	117	89% (104)	6
Myanmar	2013	99	224	65% (145)	18
India (Tamil Nadu)	2014	550	382	66% (252)	130
India (Andhra Pradesh)	2014	177	871	91% (792)	90
Myanmar	2014	212	no data	no data	53
Myanmar	2014	263	2,335	86% (2,013)	395

Of the 14 selected high-burden TB countries where the private expenditure for health was at least 45% of the total health expenditure, the most recent versions of national strategic plans for TB control were available for all but two. Of the 14 countries reviewed, the national strategic plans of 12 formally included retail drug outlet engagement primarily for referral of persons suspected of having TB (Table 5). Only Bangladesh and Indonesia had explicit statements on the need to engage their local pharmacy professional association. This review found that, over the last two decades, the role of retail drug outlets has evolved from pilot projects and guidance in global documents to formal incorporation into country plans.

**Table 5:** NTP Strategy or Action Plans

	% of private expenditure on health^a^	National strategic plan version	Private retail drug outlet engagement in strategy	Professional pharmacy association engagement in strategy^b^
Cambodia	79.5	2014–2020	X	
Afghanistan	78.8	2013–2017	X	
Nigeria	76.1	2015–2020	X	
Myanmar	72.8	2016–2020	X	
Philippines	68.4	2013–2016	X	
India	67.8	2012–2017	X	
Bangladesh	64.7	2015–2020	X	X
United Republic of Tanzania	63.7	2010–2015	X	
Pakistan	63.2	2015-2020	X	
Indonesia	61.0	2015–2019	X	X
Kenya	58.3	2015–2018	X	
Vietnam	58.1	2011–2015	X	
Uganda	55.6	2015–2020		
Democratic Republic of the Congo	46.9	2014–2017		

^a^Private expenditure on health as a percentage of total expenditure on health. WHO (2013) [18]

^b^Only if ‘pharmacy association’ was explicitly mentioned in the strategy instead of the generic term ‘professional association’

**Fig. 1:**
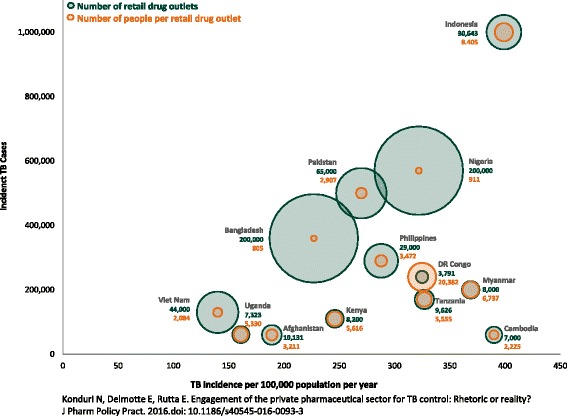
Disease burden and estimated number of retail drug outlets in 13 countries [59]

None of the 14 countries’ national strategic plans explicitly establish targets for engaging private-sector retail drug outlets or necessarily prioritize urban or rural outlets. The public health contribution of retail drug outlets to TB case finding depends on the number of retail drug outlets in the country, the population, the relative TB burden, the percentage of engaged pharmacists who refer patients, and the percentage of clients with symptoms who are successfully counseled for referral by the pharmacist. Figure 1 compares 13 of 14 countries of comparable size (excluding India) for the first three of these factors (see Additional file 3 for data sources). This figure highlights different situations within countries. Cambodia, for example has a relatively low incidence of TB cases compared to other countries but very high per capita TB burden, with one retail drug outlet serving an average of 2,225 people. Consequently, based on the 77 articles and abstracts reviewed (Additional file 2), the coverage of retail drug outlets ranged from less than 5 to 9% of the universe of retail drug outlets in a given country.

## Discussion

The findings from this review demonstrate one aspect of the evolution of PPM in TB diagnosis, care, and treatment over time. Despite the inclusion of private pharmacists in the 2001 WHO document, “*Involving private practitioners in tuberculosis control: Issues, interventions and emerging policy framework*,” evidence of the engagement of this cadre of private-sector health providers appeared slowly over the following decade. For example, in 2004, the Union World Conference on Lung Health contained no abstracts that met the inclusion criteria for this review; however, by 2014, there were 14 qualifying abstracts. A similar trend was observed in global guidance documents. The term “private sector” was mentioned in the first WHO annual TB report in 2000; however, it wasn’t until 2003 that the terms “public-private mix” and “public-private partnership” first appeared in WHO’s annual TB report. Notably, 2003 was also the first year that the annual report included the term “pharmacies.” The PPM subgroup meetings of the Stop TB Partnership showed a similar trend. While the first subgroup meeting took place in 2002, it was not until 2006 that “pharmacies/pharmacists,” “chemists,” and “seller” were included. Since that first inclusion, at least one of these terms has been in every meeting report or related document.

### Need for tailored strategies to increase national coverage

The findings of this review have several implications for the potential role of retail drug outlets in TB diagnosis, care, and treatment. The primary finding is that a variety of articles and abstracts in varying geographic locations have demonstrated that retail drug outlets are willing and able to contribute to TB control efforts, as shown by the number of referrals (Table 4). However, given the diversity of the private sector in high-TB-burden countries and the reality that NTP budgets are stretched across competing priorities, a number of considerations are warranted as countries decide whether to scale up the engagement of retail drug outlets nationwide. None of the 77 reviewed articles and abstracts mentioned or described attempts to scale up retail drug outlet engagement to increase national coverage. Pakistan had established progressive annual targets not only for engaging retail drug outlets but also for referrals and smear-positive TB cases to be detected [20].

For example, even if staff at between 10,000 and 20,000 of the drug outlets in Bangladesh or Nigeria are trained in rational antimicrobial dispensing and the referral of persons suspected of having TB, it is unlikely to have the desired public health effect, given that this comprises only 5 to 10% of the total universe of outlets. However, in most of the countries reviewed (appendix 2), the number of retail drug outlets engaged tend to be several hundred or over a thousand, which is a more manageable number to engage for quality improvement and regulatory efforts. Therefore, in Fig. 1, a large green circle (large number of outlets) means the retail drug outlet intervention is somewhat daunting and may need to rely on changing structures and incentives rather than on individual engagement, while a large brown circle (large number of clients per outlet) means that there are some useful economies of scale in engaging individual outlets. Figure 1 illustrates the reality not only for TB but also for the public health implications of irrational dispensing of antibiotics and the threat of antimicrobial resistance. Indonesia, which has a very high relative TB burden compared to Bangladesh, Nigeria, and Pakistan, has comparatively fewer retail drug outlets (30,643) that serve an average of 8,405 people per outlet, illustrating a different scenario that requires tailored strategies for maximum impact. India has an estimated 850,000 retail drug outlets. Through a public-private partnership with various entities, approximately 9% of the outlets (*n* = 75,000) in 12 selected districts across four states of India were engaged over a four-year period and accounted for nearly one-third of India’s 1.2 billion people and one-third of its smear-positive TB cases [21, 22],. This partnership reported that approximately 10 to 15% of suspected cases referred by 7,000 pharmacists over two years were found to be positive and placed on treatment. Clearly, this example illustrates not only the success of engaging retail drug outlets but also the need for steady scale up to increase national coverage. Figure 1 highlights the numbers problem that is common for PPM interventions in general but most acute for retail drug outlet interventions [23].

### Need for data on costs and number of unique referrals

This review also highlighted current gaps among the 77 articles and abstracts reviewed. For example, while many of the articles and abstracts described the potential returns of engaging retail drug outlets, none of the 12 full-text articles described the necessary inputs in terms of cost. TB programs that may need to prioritize among various PPM interventions must have an understanding of the estimated costs and cost effectiveness of each intervention [24, 25],. Such estimates, if modeled from pilot or small-scale initiatives, would allow TB programs to use a set amount of funding to determine which intervention would return the greatest output in terms of presumptive TB cases referred and ultimately confirmed. For example, one program estimated an implementation cost of $176,635 related to engaging retail drug outlets in two regions of Tanzania to inform the NTP on a scale up strategy [26]. Another factor that may impact the cost effectiveness of such an engagement is the extent to which retail drug outlets serve as the first point of care for individuals with TB-like symptoms in a given country [27, 28],. While in some countries these providers are a first point of contact for communities, in others, different points of private care may be more common, such as private clinics or hospitals. For NTPs to better prioritize interventions, an estimate of the expected cost and return is needed. At a minimum, data on the costs to engage the retail drug outlet and the resulting additional yield in new cases are needed.

As shown in Table 4, there is a great need for data on the number of unique referrals (i.e., persons suspected of having TB or TB-like symptoms) provided by retail drug outlets as opposed to an aggregate number of referrals provided by all PPM providers. Such data would allow countries to compare the relative potential and benefit of engaging various actors within the realm of PPM. Data on periods of longer than two years on the number of referred persons who eventually visited an approved diagnostic clinic and were confirmed as smear-positive TB are also needed. Further studies are needed to examine the affordability and sustainability of incentive mechanisms, such as phone credit, compared to moral persuasion [29, 30].

### Role of national pharmaceutical associations

Retail drug outlets do not operate in isolation and have linkages with wholesalers, distributors, and retail associations. While the majority of the 52 reviewed interventions primarily trained retail drug outlets to provide referrals, there are opportunities to engage other stakeholders as part of a multi-pronged intervention. A country’s national pharmaceutical association must be engaged to tap into its network of pharmacy professionals and provide policy guidance to identify educational, managerial, and regulatory approaches to engage retail drug outlets. Cambodia, India, and the Philippines, for example, have involved their local pharmacy associations significantly in advocacy and mobilization among their member networks of retail drug outlets [21, 31, 32]. In our review of the national strategic plans of selected countries, only Bangladesh and Indonesia explicitly made statements of intent to engage professional pharmacy associations. While Cambodia, India, and the Philippines have engaged professional pharmacy and retail drug outlet associations, an explicit statement was not found in any of their updated 2015–2020 national strategic plans. It cannot be assumed that the engagement of professional associations is automatic because changes in program leadership and/or fluctuations in funding levels can influence priorities. Major knowledge gaps often found among retail drug outlets, such as the etiology of the disease, awareness of public-sector programs, and referrals to accredited private clinics, can be addressed by leveraging partnerships with the national pharmacy association.

**Fig. 2:**
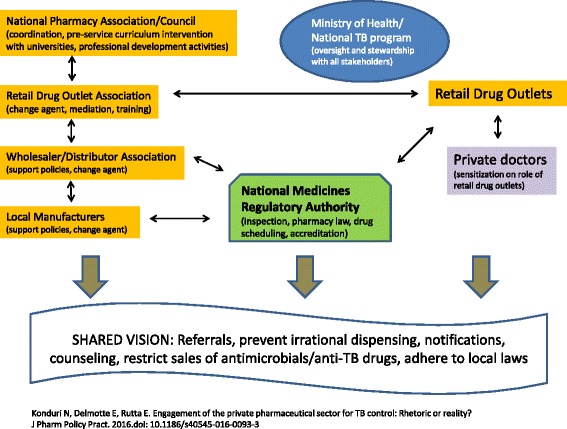
A generic PPM Pharmacy Model [59]

For PPM interventions involving retail drug outlets, the intervention design needs to consider the different players and their roles and align incentives for each stakeholder (Fig. 2). In the short term, the focus may be to train staff at retail drug outlets to identify common presenting signs and symptoms of TB and refer patients to facilities where they can be properly diagnosed and managed. However, in the long term, more stakeholders need to be sensitized to exert their role. A country’s national pharmaceutical association can work with pharmacy schools to revise their curricula to ensure that pharmacy assistants and pharmacists have the requisite knowledge on TB and the rational use of antimicrobials. Pharmacy schools are a major resource not only for any baseline assessments and evaluation activities but also for engaging pharmacy students to collect data, monitor retail drug outlet performance, recognize real-world challenges and stimulate thinking on options for policy and practice [33]. None of the 77 articles and abstracts reviewed explicitly mentioned engagement with pharmacy schools. Although there is paucity of such documented experiences, the Philippines Pharmacy Association produced an instructors’ manual for revising pharmacy school curricula to incorporate content on TB control and the role of pharmacy professionals and retail drug outlets [34]. In addition, the Indian Pharmaceutical Association involved pharmacy students to act as TB community educators [35].

### Role of national medicines regulatory authority

Among the 77 articles and abstracts reviewed, only one from Ghana documented a restriction on sales of anti-TB drugs without a formal legal ban. In countries with a large presence of local anti-TB drug manufacturers and where sales of anti-TB drugs are not legally prohibited, reaching and engaging these manufacturers and their wholesalers and distributors is a key to success. In collaboration with the national medicines regulatory authority, the NTP can work with local TB drug manufacturers to engage their sales representatives to promote the recommended WHO first-line medicines instead of loose, single TB tablets, to encourage dispensers to screen and refer those patients who present with persistent cough, and to not sell antimicrobials or anti-TB drugs until a proper diagnosis is made. Given the influence that wholesalers and distributors have on the retail sector, these actions could have a tremendous effect, particularly in countries with a large private-sector market [36]. For example, in Mumbai, India, certain retail drug outlets were accredited and recognized to serve as DOTS providers rather than perceiving this engagement as a loss of business.

The national medicines regulatory authority must be involved in relevant stakeholders meetings and training sessions involving retail drug outlets and related professional associations. Presenting a unified approach will ensure that all key stakeholders share a vision of improved early case identification and referral, reduced inappropriate antimicrobial and TB medicine sales, better contributions to overall TB notifications, and adherence to regulations and laws. In countries where an outright regulatory ban on the import and sale of anti-TB drugs is possible, consistent enforcement is key to preventing a recurrence because of the availability of anti-TB drugs in retail drug outlets [37]. In other settings, such as India, which issued a regulation in 2014 to limit over-the-counter sales of not only anti-TB drugs but also specific antimicrobials, enforcement is not sufficient, and incentives among all stakeholders must be aligned [38]. Recent systematic reviews of retail drug outlets and of the quality of pharmacy services in Asia found limited evidence of interventions related to regulatory enforcement and profit motives among private retail drug outlets [39, 40],. Therefore, it is important to place the PPM pharmacy model intervention within the larger PPM intervention package, particularly in countries with a high to moderate TB burden and a large proportion of the population receiving private-sector care [41]. It may be appropriate for referrals to be organized within private-sector providers, such as doctors, general practitioners, chest physicians, specialists, or clinics/hospitals, which may be where patients prefer to seek treatment. In most cases, the NTP may already be working with private-sector providers, such as general practitioners and hospitals.

Given the limited resources and competing priorities within the NTP, a long-term strategy could be for the Ministry of Health, particularly in countries with a large private sector to address other disease programs and invest in the PPM pharmacy model with links to the primary care system. Interventions that are comprehensive, such as Tanzania’s accredited drug dispensing outlet model, could be a good investment in the long run for TB and other diseases, such as diarrhea and malaria, and for family planning services [42].

### Limitations of this review

This review primarily focused on the role of private-sector retail drug outlets in TB control. It is possible that we may have missed some papers that did not use the terms in our search methodology. For example, community pharmacies in Bangladesh are called “village doctors” and are sensitized to refer patients suspected of having TB [43]. Because we excluded studies that were primarily focused on health-seeking behaviors of patients, we may have missed content related to the situation assessment of retail drug outlets and possible information on referrals. In addition, the relative scarcity of full-text articles on this topic compared to conference abstracts may have induced bias in the interpretation of past work involving retail drug outlets and the referral of persons suspected of having TB. The content of the abstracts varied widely in the extent of information available, and we did not have access to posters or presentations that may contain some detail on the content. The primary authors attempted to contact at least 20 selected authors of the 65 reviewed abstracts for further clarification and to seek posters or presentation files but heard back only from 6 authors. We did not perform any formative review of other Union regional conference materials or of grey literature from international development partners working with NTPs. We may have also missed presentations made at other conferences related to private-sector health care. Finally, we excluded other studies that may have documented experiences of retail drug outlets for other health conditions, such as malaria, family planning and reproductive health, and HIV. Despite these limitations, our paper has value in blending information from global and country documentation related to the objectives of this review.

## Conclusion

For the End TB Strategy to be successful, prioritizing and harnessing the power of private-sector retail drug outlets will be instrumental in accelerating the early detection and referral of the 3 million missing cases. The proposed PPM pharmacy model could become a scalable reality and make a significant contribution by harnessing both short-term solutions such as systematically engaging retail drug outlet dispensers and long-term solutions like partnerships with pharmacy schools and pharmacy associations that is long overdue in many countries. For decades, we have known about the potential of retail drug outlets but their level of engagement has not been commensurate with the TB burden and rapid growth of the private health sector. To successfully scale up PPM pharmacy models and reach ambitious targets, the international TB community must tailor interventions to the size and reach of each country’s retail drug outlet network, particularly in settings with a thriving private sector.

In addition, from a public health and pharmaceutical policy perspective, the crisis of antimicrobial resistance is unlikely to be adequately addressed through a disease-specific framework. Country authorities must recognize the community’s well-documented preference for seeking services from retail drug outlets and make a concerted effort to increase coverage of retail drug outlet engagement across the health system.

## Additional files

Additional file 1: Search terms used. (PDF 187 kb)
Additional file 2: Summary of country-level studies or interventions involving retail drug outlets. (PDF 501 kb)
Additional file 3: Data sources for Fig. 1 – comparison of retail drug outlets with corresponding TB burden and population size. (PDF 113 kb)

## Abbreviations

DOTS: Directly observed treatment short course
FIP: International Pharmaceutical Federation
NTP: National TB programs
PPM: Public-private mix
TB: Tuberculosis
USAID: United States Agency for International Development
WHO: World Health Organization
