# Supplementary material for: Engagement of the private pharmaceutical sector for TB control: rhetoric or reality?
Source: J Pharm Policy Pract. 2017 Jan 18;10:6. doi: 10.1186/s40545-016-0093-3 (PMC5241918; doi:10.1186/s40545-016-0093-3)
Supplement: Additional file 1: — Search terms used. (PDF 187 kb) [file 40545_2016_93_MOESM1_ESM.pdf]

Additional file 1: search terms used

PubMed Terms	Google Scholar Terms
TB AND private AND seller*	TB private seller
Tuberculosis AND private AND seller*	Tuberculosis private seller
TB AND private AND retail*	TB private retail
Tuberculosis AND private AND retail*	Tuberculosis private retail
TB AND private AND chemist*	TB private chemist
Tuberculosis AND private AND chemist*	Tuberculosis private chemist
TB AND private AND pharmaci*	TB private pharmacy
Tuberculosis AND private AND pharmaci*	Tuberculosis private pharmacy
TB AND private AND vendor*	TB private vendor
Tuberculosis AND private AND vendor*	Tuberculosis private vendor

The search was performed in the period November-December 2013.
