# Supplementary material for: Engagement of the private pharmaceutical sector for TB control: rhetoric or reality?
Source: J Pharm Policy Pract. 2017 Jan 18;10:6. doi: 10.1186/s40545-016-0093-3 (PMC5241918; doi:10.1186/s40545-016-0093-3)
Supplement: Additional file 2: — Summary of country-level studies or interventions involving retail drug outlets. (PDF 501 kb) [file 40545_2016_93_MOESM2_ESM.pdf]

Additional file 2: Summary of country-level studies or interventions involving retail drug outlets

*** indicates articles retrieved from PubMed
** indicates articles retrieved from Google Scholar
Articles with no asterisk are from the International Journal of Tuberculosis and Lung Disease's Union World Lung Conference Abstract book (http://www.theunion.org/what-we-do/journals/ijtld/conference-abstract-books)

# indicates abstracts with number of referrals or smear positive cases from retail drug outlets

Study	Type of Engagement	Coverage	Target providers	Result
			**Afghanistan**	
Rahman **(1)**	Intervention		• Medical officers<br>• Pharmacists<br>• Community health worker (CHW) supervisors<br>• CHWs	CHWs identify suspected TB cases, refer them for a sputum examination, and provide DOTS and follow-up during the continuation phase.
Rasooli **(2)**	Intervention		• Private hospitals<br>• Private clinics<br>• Pharmacies	845 TB suspects were identified in 169 non-NTP facilities. Among these, 498 were registered as TB suspects, and 161 of those were diagnosed with TB.
Sanaie **(3)**	Assessment	660 pharmacies; 6 provinces	• Registered pharmacies	Data has been collected through interviews with private pharmacies. Results not available in abstract.
			**Bangladesh**	
Hossain **(4)**	Intervention	17,636 participants	• Non-graduate private practitioners (e.g., village doctors and drug sellers at pharmacies)<br>• Intern doctors<br>• Factory workers	Conclusion: strengthening the linkage and regular follow-up mechanisms for participants who have been oriented would be helpful in PPM DOTS interventions.
Karim **(5)**	Intervention	322 participants	• Female community health volunteers (CHVs)<br>• Private practitioners<br>• Pharmacists<br>• Village doctors<br>• Community leaders	959 patients were diagnosed with TB.

Study	Type of Engagement	Coverage	Target providers	Result
Karim **(6)****	Assessment	1006 participants	• Village doctors<br>• Drug sellers	Most drug sellers did not refer TB suspects to the DOTS facilities at either public or private/NGO clinics for diagnosis. Although qualitative findings revealed that most drug sellers believed that public facilities might be the place for TB diagnosis and treatment, they did not refer patients to those facilities for a variety of reasons.
Rifat **(7)**	Intervention		• Female CHVs<br>• Private practitioners<br>• Physicians at academic institutions<br>• Pharmacists<br>• Village doctors	Over 5 months, a total of 1281 new sputum-positive patients were diagnosed.
Roy **(8)**	Intervention		• Female CHVs<br>• Private practitioners<br>• Pharmacists<br>• Village doctors<br>• Community leaders	Over 11 months, 7141 new sputum-positive patients were diagnosed, and of these, 6217 were from limited access areas.
Gill **(9)**	Intervention		• Clinic staff<br>• Garment workers<br>• Pharmacists<br>• HIV/AIDS workers<br>• Religious leaders	In 2011, the Smiling Sun Franchise Program treated 5378 TB cases, of which 2204 were new smear positive, 275 were smear-positive relapses, 1400 were smear-negative, and 1581 were extrapulmonary.
**Bolivia**				
Lambert **(10)***** #	Intervention	70 pharmacies	• Private pharmacies<br>• Local pharmacists association	The proportion of pharmacies selling TB drugs decreased (rifampicin: 23-11.5%; isoniazid: 16-3.1%; p<0.001), and the proportion of pharmacies referring patients seeking TB drugs to the NTP and clients increased (22-58%; p<0.0001). In the second phase, 26 of 70 pharmacies (38%) referred a total of 41 clients to the NTP for screening.
Lambert **(11)*****	Assessment	100 pharmacies	• Private pharmacies	In an anonymous patient exercise presenting with a prescription of 4 TB drugs, 25 pharmacies sold at least 1 TB drug. Of the 99 pharmacies unable to fill the whole prescription, 59 referred the client to another pharmacy, and 22 made referrals to public services.
**Burkina Faso**				
Ouedraogo **(12)** #	Intervention	339 participants	• Pharmacy personnel	339 people were trained in the detection of TB and saw 46,421 patients. 821 patients (2%) were referred to a center for the diagnosis and treatment of TB. 361 (39%) underwent a sputum culture for acid-alcohol-resistant bacillus, and of these, 17 positive cases and 1 negative case were detected.

Study	Type of Engagement	Coverage	Target providers	Result
**Cambodia**				
Bell **(13)***	Intervention, evaluation	54 participants	• Private pharmacies	Owner commitment to program goals was motivated by altruism, pragmatism, and professionalism. Of concern to participants were issues associated with patient counseling, fear of infection, and quality of care in public-sector clinics. Owners believed ongoing professional support, improved public-sector patient care, and media campaigns would strengthen their role.
Chana **(14)**	Intervention		Public and private sectors:<br>• Doctors<br>• Nurses<br>• Pharmacists<br>• Patients	The Cameroon Pharmaceutical Society and the National Tuberculosis and Leprosy Programme have used findings to map roles and develop strategies that address the needs of community pharmacies. Nine community pharmacy trainers of trainers have been trained, and protocols and checklists for case findings and referrals have been developed.
Mao **(15)**	Assessment	552 participants. Anonymous shoppers visited 273 pharmacies to validate pharmacy interview findings.	• Private doctors<br>• Pharmacies<br>• Drug sellers<br>• TB patients	Private-sector patients are generally not observed while undergoing treatment; TB drug-dispensing practices are not in accordance with NTP guidelines; and the stigma of TB is a strong deterrent for seeking treatment.
Mao **(16)**	Intervention		• All private providers	In 2011, 5024 TB suspects were referred by private providers to public DOTS faculties. Among those, 2920 visited public facilities, and 691 were diagnosed with TB.
Mao Tan **(17)**	Intervention		• Private pharmacies<br>• Private cabinets<br>• Private clinics<br>• Private laboratories	In 2007, 5660 TB suspects were identified and referred by private providers to DOTS facilities. Among those referred, 2899 visited public facilities, and 814 TB cases were treated under DOTS.
Mihalea **(18)** #	Intervention		• Pharmacies	During the first two years of the intervention, 4230 TB suspects were referred from pharmacies to DOTS centers, 3356 were screened and of those, 1769 were found to be smear positive. Interviews with TB suspects referred by pharmacies but not documented as having visited a DOTS center revealed that 94% visited a center for follow-up services.
Saly **(19)**	Assessment	398 respondents	• Clinicians<br>• Drug sellers<br>• Laboratories	Most drug sellers had good knowledge of TB symptoms, with 62% of pharmacies selling TB drugs, 42% selling TB drugs without a prescription, and 73% referring TB suspects to medical facilities.
Sokhanya **(20)**	Intervention	129 private pharmacies<br>67 private clinics	• Private pharmacies<br>• Private clinics	From July 2005 to February 2006, 454 TB suspects were identified and referred to public DOTS.

Study	Type of Engagement	Coverage	Target providers	Result
Uchiyama **(21)****	Assessment		• TB patients<br>• TB service providers<br>• Storekeepers	The prescribing practices of TB service providers were acceptable. The level of new smear-positive TB outpatients' knowledge of TB treatment was high. The storekeepers' inventory practices for TB drugs indicated a need for improvement.
**Dominican Republic**				
Kamp **(22)**	Intervention		• Community groups<br>• Public and private health providers<br>• Pharmacies<br>• Grocery shops	During a 9-month period, 236 suspects and 23 smear-positive TB cases were referred by the targeted community.
Mitchell **(23)**	Intervention	167 pharmacies	• Pharmacies	Compared to baseline, pharmacies in the intervention arm increased their spontaneous recognition of TB from 6.6% to 38.1% and were more likely to refer patients to the TB program (OR 1.6, 95% CI: 1.3-2.1 *p*=0.01). Workshops were less acceptable than detailing visits and conveyed no additional referral benefit. Unvisited pharmacies were more than twice as likely to offer antibiotics.
Mitchell **(24)****	Intervention	291 pharmacies and shops	• Pharmacies<br>• Community shopkeepers	The TB recognition and referral scores of all exposed establishments increased by an average of 2 points at post-test compared to an increase of 0.6 points among the comparison group. Attempted sales of antibiotics and palliatives declined. After 6 months, 33.1% of pharmacies and 22.7% of grocery stores reported referring patients. Seven smear-positive TB cases were diagnosed from among 70 TB suspects referred by pharmacies, but none were diagnosed among the 30 suspects referred by grocery stores.
**Ghana**				
Bonsu **(25)**	Regulatory		• Regulatory authority<br>• Healthcare professional bodies<br>• Private practitioners<br>• Private pharmacies	Restriction of anti-TB medicine sales in private pharmacies without a formal legal ban.
Bonsu **(26)**	Intervention	Selected sites in Accra metropolis only	• Personnel at health facilities and antiretroviral therapy (ART) clinics<br>• Pharmacy operators<br>• Chemical seller operators	Over 6 months, 3567 TB suspects (approximately a two-fold increase) were identified and referred for sputum examinations, and 523 new TB cases were diagnosed, including 150 from ART clinics. Pharmacies are willing partners to screen clients for TB.

Study	Type of Engagement	Coverage	Target providers	Result
Bonsu **(27)**	Intervention	An intervention and comparator sites were identified. In the interventions sites, 5 large hospitals were identified, and the various interventions were implemented to the extent possible.	• Staff at outpatient departments (unclear)<br>• Pharmacies<br>• Chemical sellers	More cases were identified from intervention sites than from comparator sites. Other health facilities have started implementing interventions on their own.
**India**				
Ananthakrishnan **(28)**	Intervention	Popular stars participated in the initiative. Five 15-minute documentaries and four short films of 1-2 minutes each have been produced.	• Private practitioners<br>• Medical students<br>• Pharmacists	These documentaries and films have been distributed widely to public health systems and serve as teaching videos during training for target groups, such as private practitioners, medical students, and pharmacists. The films focus on facts about TB, availability of quality care, etc.
Ananthakrishnan **(29)** #	Intervention	402 pharmacies	• Private pharmacies	Within the 402 pharmacies in the study, 89% of the pharmacists were aware of the symptoms of TB, and 48% were aware that TB was diagnosed with a sputum examination. While 90% of the interviewed pharmacies were dispensing anti-TB drugs, only 27% knew about the DOTS program. Almost all were willing to participate in the DOTS program. Approximately 101 TB suspects were referred from 64 pharmacies in the year following sensitization.
Ananthakrishnan **(30)**	Intervention	854 pharmacies	• Private practitioners<br>• Pharmacies<br>• Private hospitals<br>• Community volunteers<br>• Media celebrities<br>• Corporates	Approximately 5264 TB patients were registered through the PPM initiative over an 11-year period. Approximately 854 pharmacies were sensitized on TB, and of those, 11% had referred their patients for DOTS or functioned as DOT providers.
Ananthakrishnan **(31)** #	Intervention	550 participants	• Private pharmacies	550 pharmacists attended the TB orientation workshop, after which there were 382 documented and validated referrals over 15 months. Of these, 252 were chest symptomatics, and 30 were TB patients diagnosed in private-sector facilities. Among the symptomatic patients, 70% underwent sputum examinations, and 39 were sputum positive. Overall, 76 TB patients began treatment, with 68% of those being treated under the government DOTS program. 23 patients received DOT from pharmacists.
Babu **(32)** #	Intervention	84 participants	• Pharmacists	Between February and March 2012, 23 TB suspects were referred by trained pharmacists for sputum microscopy, and 8 were found to be smear positive. 7 were put on treatment.

Study	Type of Engagement	Coverage	Target providers	Result
George **(33)**	Intervention	1491 participants of which 560 were chemists	• Allopaths<br>• ISMH (practitioner of India systems of medicine and homeopathy)<br>• LTFQ (less than fully qualified provider)<br>• Chemists	Knowledge of DOTS increased, communication activities reached 538,599 people, and health-seeking behavior improved among chest symptomatics. 7030 referrals were made, and 1087 cases were notified.
Gharat **(34)**	Intervention	More than 400 pharmacists	• Pharmacists	For a national scale-up, a memorandum of understanding (MoU) was signed in April 2012 between the ministry of health and the Indian Pharmaceutical Association (IPA); this was renewed for 2 additional years in May 2013. The IPA has started training pharmacists in other states. Focused group discussions revealed that pharmacists work mainly with non-financial incentives.
Gharat **(35)**	Intervention	Over 300 participants	• Retail pharmacists	Project sites had an increased trend in case detection and patient-centric DOTS delivery. The findings of the initial projects were communicated to policy-makers in a workshop. The Revised National Tuberculosis Control Program (RNTCP) signed an MoU and formed a national core committee to review participation on pharmacies in the RNTCP. The model is now being scaled up to 3 states, followed by other states in a phased manner.
Gharat **(36)**	Intervention	126 participants	• Community pharmacists	One year after the initial training, 38 pharmacists have started delivering DOT services, and more than 50 pharmacists have referred chest symptomatic cases to government diagnostic centers. The city TB office and pharmacists keep records of the pharmacists' referred cases. All pharmacists are distributing eTB literature to patients.
Gharat **(37)**	Intervention	305 participants	• Community pharmacists	Pharmacists have become focal points for community help and have gained respect and status in the eyes of both the public and the government. Media has given visibility to the work of pharmacists. Several patients in the private sector, who would likely have been defaulters, could be converted to DOTS, and several new cases could be detected.
KK **(38)**	Intervention	438 participants	• AYUSH (Ayurveda, Yoga and Naturopathy, Unani, sidda and Homeopathy) practitioners<br>• Community pharmacists<br>• Traditional healers	Of the 438 rural health care providers (RHCPs) who were trained, 78% were AYUSH practitioners, 12% were community pharmacists, and 10% were traditional healers. Of the 902 referrals from the RHCPs, 52% were from AYUSH practitioners. The sputum positivity among the referrals was highest with the RHCPs (9%), followed by traditional healers (6%) and community pharmacists (5%). Approximately 112 RHCPs provide DOTs for 147 patients, including 82 AYUSH practitioners for 102 patients, 18 pharmacists for 21 patients, and 12 traditional healers for 18 patients. Overall, 190 sputum samples were transported by RHCPs, with a sputum positivity of 4.2%.
Kaipilyawar **(39)** #	Intervention	60 participants	• Private chemists	Of the 60 chemists and druggists who were trained, 32 (53%) referred at least one client. 117 suspects were referred by chemists, and 104 (89%) of those went to and were served at designated microscopy centers. Of the 104 referrals, 6 cases of TB were detected, which was an increase of 2.4% in case detection for 3 centers.

Study	Type of Engagement	Coverage	Target providers	Result
Panibatla **(40)** #	Intervention	475 participants	• Pharmacists and rural health care providers (RHCPs)	115 of the 177 trained pharmacists (60%) are actively participating in the project and in the NTP. From February 2013 to January 2014, pharmacists referred 871 TB-symptomatic persons for testing, and approximately 91% (792/871) of those underwent testing. Approximately 11% (90/792) of those tested were diagnosed with TB. Approximately 10 pharmacists provided DOTS to 11 people undergoing CAT-1 treatment. Among the 10 people with TB who received treatment from pharmacists, 5 had completed the full course and were cured, and 4 had completed the intensive phase and were declared as negative at the first follow-up. The remaining two are under treatment. Pharmacists are also recognized as DOTS providers by the NTP. Referrals from pharmacists on average accounted for 5% per month among the total symptomatic tested patients in the TB units.
Rajeswari **(41)*****	Assessment	300 pharmacies	• Private pharmacies	2800 prescriptions were dispensed monthly by pharmacies. Doctors' prescriptions were for durations of several months, but half of the patients bought drugs one dose at a time for self-administration. 95% of pharmacists were not aware of the existence of the TB program, and 97% were willing to learn and contribute to TB control.
Vijayan **(42)**	Intervention	More than 70 project pharmacists have cured 225 patients, and the positive case detection rate among the referred cases was 16%. The IPA advocated to the RNTCP to scale up this initiative to 500,000 pharmacies	• Community pharmacists	The RNTCP signed an MoU with the IPA and the All India Organization of Chemists and Druggists to carry forward pharmacist engagement throughout India. Three states were selected as pilot states.
Vijayan **(43)**	Assessment	Mapping of private practitioners and chemists in 15 wards of Mumbai, covering 8.9 million people comprising approximately 85% of the slum population.	• Private practitioners<br>• Chemists	8519 providers were identified including chemists (1 per 100,000 population), with a total of 276 different degrees, diplomas, and certifications; among these, unclassified accounted for 233 types of degrees. Among the total mapped, qualified providers were 1,852 (22%) less than fully qualified (LTFQ) were 3,416 (40%), chemist 2,763 (32%) and unclassified 488 (6%). Analysis of slum/non-slum provider distribution was done for two wards, and the proportion of LTFQs was significantly higher in non-slum areas than in slum areas in these two wards. Strategies for early detection of TB will require extensive utilization of the LTFQ provider groups.
Yellappa **(44)**	Assessment		• Private-sector providers of TB care<br>• Patients on private TB treatment<br>• Private pharmacies and labs	Approximately 30% of lab and pharmacy revenues from TB diagnostic tests and anti-TB medicines were paid to the referring private practitioners (PPs). PPs have a significant financial incentive to retain TB patients. However, it may be feasible to provide sufficient financial incentives for PPs to report and refer TB patients to free-of-charge government TB services earlier in the course of treatment.
**Kenya**				

Study	Type of Engagement	Coverage	Target providers	Result
Kamule **(45)**	Intervention		• Private practitioners<br>• Pharmacies<br>• Herbalists	Liaisons with private providers resulted in referrals for those who continued to seek care for an ailing cough.
Sitienei **(46)****	Assessment	176 facilities and outlets	• Health care facilities<br>• Medicine retail outlets	Investigators found that 96.8% (n=90) of private pharmacies that were visited had or claimed to have one or more anti-TB medicines. Surprisingly, only 14% of the retail medicine outlets that were visited stocked and sold the full course of anti-TB medicines.
Sitienei **(47)**	Intervention		• Private-sector and chemists<br>• HIV, STI clinics<br>• Other providers	In 2012, 102,514 cases were reported through contact investigation processes. Chemists, among 5 other providers, contributed to 7% referrals of contacts suspected of having TB.
**Lao PDR**				
Glish **(48)**	Intervention	600 participants	• Pharmacists	More than 600 referral cards were distributed in 6 months, and 21% were collected at a health facility. Among the successful referrals, TB case detection increased from 12% to 28%.
**Myanmar**				
Aung **(49)**	Assessment	97 participants	• Drug sellers	35% of drug shops had anti-TB drugs, and 38% had sold anti-TB drugs during the last month. Approximately 79.4% had low knowledge of the signs and symptoms of TB, and 97.9% had low knowledge of anti-TB treatments. 21.6% of drug sellers said they referred TB suspect cases to the nearest health center. Drug sellers would like to attend short trainings on TB conducted by health personnel during the weekends.
Htay **(50)** #	Intervention	99 drug shops	• Registered drug shops	From July to December 2012, 56 of 99 drug shops referred at least one TB suspect. In total, 224 TB suspects were referred, and 145 (64.7%) visited the township health center. The smear positivity rate was 14.8% for adults. Referred cases by drug sellers accounted for 11.9% of smear-positive pulmonary TB and 9.6% of all forms of registered TB in the township.
Nwe **(51)** #	Intervention	212 participants	• Drug sellers	Of the 365 registered drug sellers, 212 (an average of 62%) participated in the program. Of these 212, 71 (an average of 34%) referred at least one presumptive TB case to a diagnostic facility. 251 presumptive TB patients visited township health facilities after referrals from drug sellers. Of these, 53 smear-positive cases (an average of 20%) were detected and 77 (an average of 31%) were treated for TB. According to drug sellers, one problem they experienced is that some presumptive TB patients didn't visit diagnostic centers due to time and the cost of transportation.
Swe **(52)** #	Intervention	408 pharmacies	• Registered pharmacies	263 of 480 recruited pharmacies (55%) participated in TB active case finding activities. From July 2012 to December 2013, active pharmacies referred 2335 presumptive TB patients to diagnosis and treatment centers. 86% (2013) of the referred clients underwent diagnostic procedures, and of those, 19.6% (395) had bacteriologically

Study	Type of Engagement	Coverage	Target providers	Result
				confirmed TB. 18.9% (306) of clients that were smear negative underwent further evaluations, and 179 of those were clinically diagnosed with TB. 531 of 574 diagnosed TB patients were registered at Sun Quality Health (SQH) clinics for treatment. The prevalence of bacteriologically confirmed TB among patients with presumptive TB was higher in pharmacies (19.6%) than in SQH clinics (11% in 2013).The low participation of pharmacies was driven by social stigma and time constraints. Loss to follow-up among referred patients was attributed to poor knowledge about TB among patients and inadequate diagnostic facilities.
Win **(53)**	Intervention	87 participants	• Interpersonal communicators<br>• Trained pharmacies	The percentage changes of TB cases that were detected varied from a 61% increase to a 154% increase. One township showed a 3% decrease. The contributions by this linkage with the private sector range from 21% to 68% of total detected TB cases in each township.
Win **(54)**	Intervention	586 participants	• Interpersonal communication agents<br>• Staff in pharmacies	Population Services International's intervention resulted in an average increase of new smear-positive cases and all forms case notification of 9%. 1859 SQH TB patients were identified through interpersonal communications (88%) and pharmacy (12%) activities during the project period.
**Nepal**				
Hurtig **(55)*****	Assessment	98% of private drug retailers in the area	• Private drug retailers	65% of pharmacies had sold anti-TB medicines during the last month. 88% said that most TB patients were of low socioeconomic status and rarely bought more than a one-week supply of medicine at a time. 16% reported that TB patients usually returned to buy full course of medicine. 38% of pharmacies with no doctors had sold anti-TB medicines compared to 71% of those with doctors.
**Nigeria**				
Asieba **(56)**	Intervention	63 participants	• Community pharmacists	63 community pharmacists were enrolled in the program, 1758 referrals for TB were made, 298 smear-positive clients were detected and placed on TB therapy, and 112 persons were supported on treatment. The TB sputum positively rate among referred clients was 5.9%. The participating pharmacists offered their services and time without charge as part of their commitment to improving public health.
Okolo **(57)**	Assessment	150 participants	• Community pharmacists	Of 129 responses, 86.8% stocked anti-TB medicines, and 67.2% had dispensed those medicines in the month prior to the survey. 43.3% dispensed medicines without a doctor's prescription. When they see cases suspected to be TB, 7% would place the patient on routine anti-TB medicines, while 93% would refer the patient to a hospital (77.7%), laboratory (9.8%), or both (3.6%).
Onyeneho **(58)****	Assessment	422 participants	• Patent medical vendors<br>• Principal officers of patent medicine vendor association<br>• Community leaders	90% of patent medicine vendors indicated that they would be ready to cooperate with the NTP if they were trained. 73% had treated patients with prolonged cough in the course of their career. However, 48% did not know the cause of TB. Only 3% had attended a training on TB control. 66% had completed at least 12 years of schooling with a secondary school certificate. 80% of community leaders were happy with the work of patent medicine vendors.

Study	Type of Engagement	Coverage	Target providers	Result
**Pakistan**				
Hadi **(59)**	Assessment	150 participants	• Chemists	Only 22% of providers have heard of the NTP, and only 2% have received any kind of TB training in the past two years. The NTP designed an innovative approach to training and established referral linkages between retail chemists and TB DOT centers to improve case detection and appropriate dispensing of TB medicines.
Khalid **(60)**	Assessment	N/A	• 132 patients with suspected multidrug resistant TB presenting to Karachi's largest public-sector hospital	Fluoroquinolone resistance was observed in an alarmingly high proportion of multidrug resistant TB cases. Engaging private sector providers and stringent pharmacy regulations are required to prevent an extensively drug-resistant TB epidemic.
Olarewaju **(61)**	Assessment	47 participants	• Community pharmacists	80.9% of community pharmacists interviewed were seeing TB suspects and had good knowledge of TB etiology (100%) and airborne routes of transmission (70%). 90% did not know the TB treatment duration and standardized drug regimen for adults (93%) or children (97.9%). 40.4% regarded sputum microscopy as the best test to confirm a diagnosis of pulmonary TB. 99% had no previous training on standard guidelines on TB control from the national program.
**Philippines**				
Carandang **(62)**	Assessment	95 participants	• Community pharmacists	Phase 1: Most pharmacists and staff provided insufficient information on anti-TB medicines. Phase 2: Some pharmacists provided only a little information needed by the simulated patient. Phase 3: A mean of 6.15 was interpreted as "often" and showed that pharmacist- and staff-provided counseling on anti-TB medicines was limited to common information, such as the name of the medication, the indication, and the dosage.
Costello **(63)** #	Intervention		• Pharmacy personnel	Within 10 months, pharmacy personnel served 7000 clients and referred one-third of these to DOTS facilities.
Villanueva **(64)** #	Intervention with control group	1170 participants	• Pharmacy personnel	Pharmacy DOTS Initiative (PDI) pharmacies served 3979 TB-related clients and referred 1550 clients to DOTS centers, 575 of whom accessed the centers. 169 were diagnosed with TB, and 83 were smear-positive. Contributions to the local case detection rate ranged from 0.35% to 7.69%. Mystery shopper studies revealed statistically significant differences between PDI and non-PDI pharmacies regarding adherence to not dispensing without a prescription, greater knowledge on TB DOTS, and being more likely to refer patients to DOTS centers.

Study	Type of Engagement	Coverage	Target providers	Result
Villanueva **(65)** #	Intervention	119 pharmacies, local drugstore owners, pharmacist associations, and government partners were engaged to provide advocacy support to mitigate self-medication practices.	Trained:<br>• Pharmacies<br>Engaged to provide advocacy support:<br>• Drugstore owners<br>• Pharmacist associations<br>• Government partners	There were 942 TB symptomatic cases identified by engaged pharmacies among their clients who were referred to DOTS facilities.
**Tanzania**				
Mahamba **(66)**	Assessment	530 participants	• Health care workers	Pediatric TB guidelines were implemented in all health facilities, and 49 trainers and 481 health care workers were trained.<br>Conclusions: Future work should also take advantage of existing community innovations (i.e., use of drug sellers, traditional healers, and community-based distributors) to improve case detection.
Mugyabuso **(67)**	Intervention (TB-HIV context)		Training:<br>• Sputum fixers<br>• School health project leaders<br>Involved:<br>• Traditional healers<br>• Drug sellers<br>• Individual communities' own resource personnel	By December 2011, traditional healers and drug sellers identified 312 patients as suspect, and among these, 21.5% were diagnosed with TB.
Mwangomale **(68)**	Intervention	397 participants	• Community members, such as traditional healers<br>• Former TB patients<br>• Private drug sellers	From 2009 to 2011, there was an increase of TB cases in the Kisarawe district, while TB case notification decreased nationally. Results prompted a scaling up of community interventions to 35 districts in PATH-supported regions. Data indicate that the results of the 6-month period after scale-up are comparable to the full year prior, with nearly the same results achieved in half the time.
Tarimo **(69)**	Intervention		• Accredited drug dispensing outlets and pharmacy dispensers	95% of dispensers knew that persistent cough is a symptom of TB, but only 30% were aware of DR-TB.
**Thailand**				
Jittimanee **(70)**	Assessment		• Patients<br>• Local health care staff	All urban patients started seeking care from pharmacies when their symptoms didn't resolve because pharmacies were closer to home and had no waiting times. Rural patients visited hospitals or private clinics.<br>Conclusion: An improving referral role of pharmacies and primary health care units should be considered.

Study	Type of Engagement	Coverage	Target providers	Result
Thamsuwan **(71)**	Intervention		• Unclear: "Private agencies and CSO have received grants from TGF to implement activities for TB control"	From 2011 to 2013, the project screened 1,031,616 people and referred 5318 people with symptoms indicative of TB for diagnosis. Of these, 424 cases were confirmed. **Success is also attributed to the collaboration of the Pharmacists Association and 83 drug store members in Bangkok who helped screen cases with suspicious symptoms who went to the drug store for cough medicine and referred them for formal diagnosis and care.**
**Vietnam**				
Lonnroth **(72)***** #	Intervention	Questionnaire survey of 150 private pharmacies; positive interviews with 16 pharmacists	• Private pharmacists	A simple referral form was thought to be useful by 72% of pharmacists. Pharmacists identified a number of risks associated with referring TB suspects to the NTP, including clients turning to another pharmacy, clients criticizing the pharmacists if they do not have TB, or client remaining untreated because they are not able to visit the NTP. 39% of eligible pharmacies referred at least 1 client. At least 373 suspects were referred, 149 were tested, and 10 were diagnosed with TB.
Lonnroth **(73)*****	Assessment	147 private pharmacies	• Private pharmacies	18% of interviewees identified TB as a possible diagnosis for a fictitious case with fever and cough that persisted for 4 weeks. 58% reported selling anti-TB drugs often or sometimes. Interviewees estimated that 1.3 persons on average had bought anti-TB drugs during the last 4-week period and 24% had bought anti-TB drugs without a prescription.
Neukom **(74)**	Assessment	508 participants	• People with a long-lasting cough	A quarter of respondents (25%) did not seek any care for their TB symptoms. Among those seeking care, the majority (73.4%) sought care first in pharmacies, and 80% continued to a second source of care, with private clinics being the most common (44.7%) followed by provincial and district public hospitals.<br>Conclusion: This indicates an important role for private-sector involvement in increasing TB case detection in Vietnam. Boosting provider skills to detect and refer TB cases should be a key part of this strategy.
Pham **(75)**	Assessment	98 outlets	• Public-sector general hospitals and communal health posts<br>• Private-sector pharmacies and clinics	Anti-TB drugs were available in private pharmacies, clinics, and some general hospitals. PPM approaches should be expanded and strengthened to ensure adherence to NTP treatment guidelines.
Quy **(76)****	Intervention	508 participants	• Private physicians<br>• Private pharmacies<br>• Lung specialists working at the regional public TB hospital who also had a private practice	1549 TB suspects were referred, of whom 1090 (70%) actually went to the NTP for a sputum examination. 569 cases were detected through referrals or notifications, of whom 45% were new sputum smear-positive cases. The case detection of sputum smear-positive cases in PPM districts increased by 18% compared with the previous year, while a slight decrease occurred in the control districts.

Study	Type of Engagement	Coverage	Target providers	Result
Vu **(77)*****	Assessment	138 private pharmacies	• Health care providers in private pharmacies	In the simulated patient method and interview, 46% and 55% of health care providers, respectively, referred the simulated patient to a TB care facility. 42% of the health care providers identified suspected TB from a fictitious case on paper, and 27% were aware that the NTP provided free treatment. Pharmacies with Good Pharmacy Practice (GPP) certification were less likely to refer simulated patients to TB facilities than were non-GPP pharmacies.

Bibliography

1. Rahman MD. BRAC community-based TB control program in Afghanistan and community based TB DOTS. In Abstract Book: 37th World Conference on Lung Health of the International Union Against Tuberculosis and Lung Disease; 2006; Paris: Int J Tuberc Lung Dis. p. S264.

2. Rasooli M, Delawer F, Habib H, Andar H. Identifying all tuberculosis cases detected in the health system: a new approach to revisit the case detection rate. In Abstract Book: 41st World Conference on Lung Health of the International Union Against Tuberculosis and Lung Disease; 2010; Berlin: Int J Tuberc Lung Dis. p. S213.

3. Sanaie AH, Ahmadzai H, Huseynova S, Nuzhat L. Role of private pharmacies in central region of Afghanistan. In Abstract Book: 38th World Conference on Lung Health of the International Union Against Tuberculosis and Lung Diseas; 2007; Cape Town: Int J Tuberc Lung Dis. p. S275.

4. Hossain Z, Ahmed J, Rashid SM, Kisha BB, Islam N. Involving all care providers: BRAC's experience in TB control. In Abstract Book: 40th World Conference on Lung Health of the International Union Against Tuberculosis and Lung Disease; 2009; Cancun: Int J Tuberc Lung Dis. p. S340.

5. Karim R, Islam MA, Haque B, Alamin M, Kayum M, Begum I, et al. Private public mix DOTS in urban areas of Bangladesh: BRAC experience [abstract]. In 36th Union World Conference on Lung Health; 2005; Paris: Int J Tuberc Lung Dis. p. S219.

6. Karim F, editor. Socioeconomic Divides inTuberculosis Control: Study of Smear-Positive TB Prevalence, Care Seeking Behavior and Role of Informal Healthcare Providers Dhaka: BRAC; 2009.

7. Rifat M, Mahmud MH, Haque B, Islam MA, Ahmed F. Urban DOTS in Bangladesh. In Abstract Book: 36th World Conference on Lung Health of the International Union Against Tuberculosis and Lung Disease; 2005; Paris: Int J Tuberc Lung Dis. p. S229.

8. Roy B, Khan MH, Begum I, Islam MA, Barua MK. Reaching targets through community based DOTS by mobilizing society and maintaining quality of DOTS services in rural Bangladesh. In Abstract Book: 36th World Conference on Lung Health of the International Union Against Tuberculosis and Lung Disease; 2005; Paris: Int J Tuberc Lung Dis. p. S229.

9. Gill Z, Islam A, Veskov D, Amin K. Linking public, private and civil society networks to strengthen tuberculosis control in Bangladesh. In Abstract Book: 43rd World Conference on Lung Health of the International Union Against Tuberculosis and Lung Disease; 2012; Kuala Lumpur: Int J Tuberc Lung Dis. p. S387.

10. Lambert ML, Delgado R, Michaux G, Vols A, Speybroeck N, Van der Stuyft P. Collaboration between private pharmacies and national tuberculosis programme: an intervention in Bolivia. Trop Med Int Health. 2005; 10(3).

11. Lambert ML, Delgado R, Michaux G, Vols A, Speybroeck N, Van der Stuyft P. Tuberculosis control and the private health sector in Bolivia: a survey of pharmacies. Int J Tuberc Lung Dis. 2004; 8(11).

12. Ouedraogo. Research in Additional Case Detection of Tuberculosis in Pharmacies in Burkina Faso: Acceptability by Principal Players and Lessons Learned. In Abstract Book: 44th World Conference on Lung Health of the International Union Aaginst Tuberculosis and Lung Disease; 2013; Paris: Int J Tuberc Lung Dis. p. S393.

13. Bell CA, Eang MT, Dareth M, Rothmony E, Duncan J, Saini B. Provider perceptions of pharmacy-initiated tuberculosis referral services in Cambodia, 2005-2010. Int J Tuberc Lung Dis. 2012; 16(8): p. 1086-1091.

14. Chana R, Abong B. Engaging Cameroon pharmacists for early tuberculosis case finding and rational use of tuberculosis medicines. In Abstract Book: 44th World Conference on Lung Health of the International Union Aaginst Tuberculosis and Lung Disease; 2013; Paris: Int J Tuberc Lung Dis. p. S39.

15. Mao TE, Kong BN, In S, Perla G. Private sector TB services in Cambodia. In Abstract Book: 36th World Conference on Lung Health of the International Union Against Tuberculosis and Lung Disease; 2005; Paris: Int J Tuberc Lung Dis. p. S66.

16. Mao TE, Team BK, Tonsing J. How can Stop TB Partners assist countries in restricting misuse of tuberculosis drugs and technologies? The Cambodia experience. In Abstract Book: 43rd World Conference on Lung Health of the International Union Against Tuberculosis and Lung Disease; 2012; Kuala Lumpur: Int J Tuberc Lung Dis. p. S4.

17. Mao Tan E, Team Bak T, Seak K, Mihalea H, Samith H, Ken V, et al. Public-private mix for TB control can increase TB case detection in Cambodia. In Abstract Book: 39th World Conference on Lung Health of the International Union against Tuberculosis and Lung Disease; 2008; Paris: Int J Tuberc Lung Dis. p. S256.

18. Mihalea H, Team B, Mao D, Nguon S, Hou S, Sugiyama T. Use of pharmacies as a source of referral: a PPM-DOTS pilot in Cambodia. In Abstract Book: 39th World Conference on Lung Health of the International Union against Tuberculosis and Lung Disease; 2008; Paris: Int J Tuberc Lung Dis. p. S287.

19. Saly S, Okada K, Uchiyama Y, Miura T, Kisman K, Eang MT. TB knowledge, attitudes, and practices (KAP) of private practitioners in clinics, pharmacies and laboratories in Phnom Penh, Capital City of Cambodia. In Abstract Book: 36th World Conference on Lung Health of the International Union Against Tuberculosis and Lung Disease; 2005; Paris: Int J Tuberc Lung Dis. p. S217.

20. Sokhanya I, Saly S, Sugiyama T, Hara S, Samith H, Yann Y, et al. Introducing a PPM-DOTS model in Phnom Penh, Cambodia. In Abstract Book: 37th World Conference on Lung Health of the International Union Against Tuberculosis and Lung Disease; 2006; Paris: Int J Tuberc Lung Dis. p. S205.

21. Uchiyama Y, Mao TE, Okada K, Chay S, Kou Soum M, Leng C. An assessment survey of anti-tuberculosis drug management in Cambodia. Int J Tuberc Lung Dis. 2006; 10(2): p. 153-159.

22. Kamp AS, Orejel Juarez I, Morrobel AL, Marcelino B. Dominican Republic: partnering with CBOs and shopkeepers to improve TB case detection in urban slums. In Abstract Book: 42nd World Conference on Lung Health of the International Union Against Tuberculosis and Lung Disease; 2011; Lille: Int J Tuberc Lung Dis. p. S63.

23. Mitchell EM, Perez-Then E, Baez J, Orejel-Juarez I, Morrobel AL, Marcelino B, et al. Evaluation of two strategies to improve referral of TB suspects by pharmacies in Santo Domingo. In Abstract Book: 42nd World Conference on Lung Health of the International Union Against Tuberculosis and Lung Disease; 2011; Lille: Int J Tuberc Lung Dis. p. S134.

24. Mitchell E, Perez-Then E, Orejel-Juarez I, Baez J, Gonzales F, Morrobel AL, et al. Effectiveness of Interventions to Increase Referral of Clients Exhibiting TB Symptoms by Pharmacies and Corner Stores in Santo Domingo, Domincan Republic. The Open Infectious Diseases Journal. 2013;: p. 47-53.

25. Bonsu F. Tuberculosis drugs are hard to find in private pharmacies in Ghana: what makes this possible?. In Abstract Book: 43rd World Conference on Lung Health of the International Union Against Tuberculosis and Lung Disease; 2012; Kuala Lumpur: Int J Tuberc Lung Dis. p. S3.

26. Bonsu F, Ohene SA, Seckey A, Danso S, Mensah G, Ahiabu MA, et al. Using targeted approaches to improve TB case detection in Accra, Ghana. In Abstract Book: 42nd World Conference on Lung Health of the International Union Against Tuberculosis and Lung Disease; 2011; Lille: Int J Tuberc Lung Dis. p. S340.

27. Bonsu F, Nortey H, Sackey A, Danso S, Afutu K, Ahiabu M, et al. Using Innovative approaches to increase tuberculosis case detection in Ghana. In Abstract Book: 43rd World Conference on Lung Health of the International Union Against Tuberculosis and Lung Disease; 2012; Kuala Lumpur: Int J Tuberc Lung Dis. p. S17.

28. Ananthakrishnan R, Augusteen S, Karunakaran N, Krishnan N. Using film and involving film celebrities for tuberculosis messaging and creating tuberculosis awareness: a report from Tamil Nadu, India. In Abstract Book: 44th World Conference on Lung Health of the International Union Aaginst Tuberculosis and Lung Disease; 2013; Paris: Int J Tuberc Lung Dis. p. S174.

29. Ananthakrishnan R, Krishnan N, Auguesteen S. Involving private pharmacies in urban DOTS program: preliminary report form Chennai. In Abstract Book: 41st World Conference on Lung Health of the International Union Against Tuberculosis and Lung Disease; 2010; Berlin: Int J Tuberc Lung Dis. p. S213.

30. Ananthakrishnan R, Krishnan N, Augusteen S. A public private mix partnership model for augmenting TB control efforts in Chennai, India. In Abstract Book: 42nd World Conference on Lung Health of the International Union Against Tuberculosis and Lung Disease; 2011; Lille: Int J Tuberc Lung Dis. p. S137.

31. Ananthakrishnan R, Sujatha P, Augusteen S, Krishnan N, Prasad S. Engaging private pharmacies in the Public-Private Partnership for TB Control in Chennai city, India. In Abstract Book: 45th World Conference on Lung Health of the International Union Against Tuberculosis and Lung Disease; 2014; Barcelona. p. S145-S146.

32. Babu ER, Chadha S, Mohanty S, Kishore K, Kumar A, Gharat M. Involvement of pharmacists in tuberculosis care and control: experience from Maharashtra and Tamil Nadu States of India. In Abstract Book: 43rd World Conference on Lung Health of the International Union Against Tuberculosis and Lung Disease; 2012; Kuala Lumpur: Int J Tuberc Lung Dis. p. S134.

33. George O, Ganesan R, Subramaniem S, Cherian S, Washington R, Suryakanth MD, et al. Initiative to engage private providers in the Revised National Tuberculosis Control Programme to improve case finding and holding in Karnataka urban slums. In Abstract Book: 43rd World Conference on Lung Health of the International Union Against Tuberculosis and Lung Disease; 2012; Kuala Lumpur: Int J Tuberc Lung Dis. p. S271.

34. Gharat M. Why are profit-driven pharmacies motivated to contribute to tuberculosis case detection and treatment? In Abstract Book: 44th World Conference on Lung Health of the International Union Aaginst Tuberculosis and Lung Disease; 2013; Paris: Int J Tuberc Lung Dis. p. S38.

35. Gharat M, Sheth PD, Prasad S, Vijayan S. Engaging retail pharmacists as partners in tuberculosis program: the Indian experience. In Abstract Book: 43rd World Conference on Lung Health of the International Union Against Tuberculosis and Lung Disease; 2012; Kuala Lumpur: Int J Tuberc Lung Dis. p. S4.

36. Gharat M, Vaidya R, Prasad S, Sheth P, Salave J. Engaging community pharmacists in DOTS programme for TB control: public-private initiative in Mumbai, India. In Abstract Book: 42nd World Conference on Lung Health of the International Union Against Tuberculosis and Lung Disease; 2011; Lille: Int J Tuberc

Lung Dis. p. S328.

37. Gharat M, Prasad S. Community involvement by community pharmacists in India for TB care and control. In Abstract Book: 45th World Conference on Lung Health of the International Union Against Tuberculosis and Lung Disease; 2014; Barcelona. p. S144-S145.

38. K K, Ramya A, Das A, Krishnan N. Engaging the Rural Health Care Providers (RHCPS) in TB control: report from Tamilnadu, Southern India. In Abstract Book: 45th World Conference on Lung Health of the International Union Against Tuberculosis and Lung Disease; 2014; Barcelona. p. S244.

39. Kaipilyawar S. India: monitoring and evaluation of chemist identification and referral of possible tuberculosis cases. In Abstract Book: 44th World Conference on Lung Health of the International Union Aaginst Tuberculosis and Lung Disease; 2013; Paris: Int J Tuberc Lung Dis. p. S34.

40. Panibatla V, Banavaliker J, Prasad S. Pharmacists and rural health-care providers in TB care and control, including all stakeholders. In Abstract Book: 45th World Conference on Lung Health of the International Union Against Tuberculosis and Lung Disease; 2014; Barcelona. p. S241-S242.

41. Rajeswari R, Balasubramanian R, Bose MS, Sekar L, Rahman F. Private pharmacies in tuberculosis control - a neglected link. Int J Tuberc Lung Dis. 2002; 6(2): p. 141-173.

42. Vijayan S, Gharat M, Prasad S, Sheth P. Achieving universal access through engaging community pharmacists in India. In Abstract Book: 43rd World Conference on Lung Health of the International Union Against Tuberculosis and Lung Disease; 2012; Kuala Lumpur: Int J Tuberc Lung Dis. p. S228.

43. Vijayan S, Khetarpal M, Datta M, Syed I, Chopra R, Sugendran P, et al. Chemists and less-than-fully-qualified providers dominate Mumbai's health provider universe. In Abstract Book: 45th World Conference on Lung Health of the International Union Against Tuberculosis and Lung Disease; 2014; Barcelona. p. S146-S147.

44. Yellappa V, Rao N. Incentives for tuberculosis care in the private sector in India: a qualitative study. In Abstract Book: 45th World Conference on Lung Health of the International Union Against Tuberculosis and Lung Disease; 2014; Barcelona. p. S251.

45. Kamule. Early identification strategies of potential tuberculosis suspects in communities of Western Kenya. In Abstract Book: 41st World Conference on Lung Health of the International Union Against Tuberculosis and Lung Disease; 2010; Berlin: Int J Tuberc Lung Dis. p. S141.

46. Sitienei JK, Gitau SK. Prescription and Dispensing of TB Medicines: A Survey of Practices in the Private Health Sector of Kenya, July 2007. Nairobi: Management Sciences for Health; 2007 [cited 2013 December. Available from: http://www.popline.org/node/198662.

47. Sitienei J, Ong'ang'o J, Hillary K. Lessons learnt from standardizing approaches to contact investigation in Kenya. In Abstract Book: 43rd World Conference on

Lung Health of the International Union Against Tuberculosis and Lung Disease; 2012; Kuala Lumpur: Int J Tuberc Lung Dis. p. S46.

48. Glish L, Sinhradsvong L, Phanalasy S, Chanthalangsy P, Stankard P, Gray R. Tuberculosis case detection via private pharmacies in Lao PDR. In Abstract Book: 44th World Conference on Lung Health of the International Union Aaginst Tuberculosis and Lung Disease; 2013; Paris: Int J Tuberc Lung Dis. p. S339.

49. Aung ST, Lwin TD, Aung PP, Saw S, Han W, Maung TM, et al. Dispensing anti-TB drugs among pharmacies in selected townships, Myanmar. In Abstract Book: 42nd World Conference on Lung Health of the International Union Against Tuberculosis and Lung Disease; 2011; Lille: Int J Tuberc Lung Dis. p. S327.

50. Htay HH, Min Min KT, Swe Swe T, Thura M, GH , Nishiyama H. Feasibility and impact of referral of tuberculosis suspects by drug sellers in an urban township, Myanmar. In Abstract Book: 44th World Conference on Lung Health of the International Union Aaginst Tuberculosis and Lung Disease; 2013; Paris: Int J Tuberc Lung Dis. p. S340.

51. Nwe T, Tin Min Min K, Thein S, G H, Nishiyama H, Okada K, et al. Private-Public Mix (PPM) of referral of TB symptomatic by drug sellers in four townships of Myanmar. In Abstract Book: 45th World Conference on Lung Health of the International Union Against Tuberculosis and Lung Disease; 2014; Barcelona. p. S165.

52. Swe P, Zarni S, Win K. Involvement of informal health care providers, pharmacies, in population services international Myanmar's TB control activities. In Abstract Book: 45th World Conference on Lung Health of the International Union Against Tuberculosis and Lung Disease; 2014; Barcelona. p. S159-S160.

53. Win AN, Linn AK, Swe P, Gary M. Improving tuberculosis case detection in Mandalay region by establishing innovative links to private sector providers. In Abstract Book: 44th World Conference on Lung Health of the International Union Aaginst Tuberculosis and Lung Disease; 2013; Paris: Int J Tuberc Lung Dis. p. S395.

54. Win AN, Lwin M, Swe P, Thu M, Boxshall M. Improving tuberculosis case detection in Myanmar through intensified case finding in urban slum areas. In Abstract Book: 44th World Conference on Lung Health of the International Union Aaginst Tuberculosis and Lung Disease; 2013; Paris: Int J Tuberc Lung Dis. p. S346.

55. Hurtig AK, Pande SB, Baral SC, Porter JD, Bam DS. Anti-tuberculosis treatment in private pharmacies, Kathmandu Valley, Nepal. Int J Tuberc Dis. 2000; 4(8): p. 730-736.

56. I A, Omeh O, Oqua D, Adesina A, Agada P, Adeboga O, et al. Promoting tuberculosis case findings in the community through Public-Private partnership. In Abstract Book: 45th World Conference on Lung Health of the International Union Against Tuberculosis and Lung Disease; 2014; Barcelona. p. S165-S166.

57. Okolo CO. TB patients' patronage of community pharmacies in the DOTS era: a survey of Lagos State of Nigeria. In Abstract Book: 36th World Conference on Lung Health of the International Union Against Tuberculosis and Lung Disease; 2005; Paris: Int J Tuberc Lung Dis. p. S209.

58. Onyeneho NG, Chukwu JN. Is There a Role for Patent Medicine Vendors in Tuberculosis Control in Southern Nigeria? J Health Popul Nutr. 2010; 28(6): p. 567-577.

59. Hadi H, Qadeer E, Memon FN, Bukhari NI, Kiana A, Abdussamad , et al. Harnessing the popularity of chemists in high-burden tuberculosis settings in Pakistan's four urban areas.. In Abstract Book: 44th World Conference on Lung Health of the International Union Aaginst Tuberculosis and Lung Disease; 2013; Paris: Int J Tuberc Lung Dis. p. S38.

60. Khalid N, Zaidi A, Rizvi N, Haseeb A, Malik A, Khowaja S, et al. Emergence of fluoroquinolone resistance amongst multidrug-resistant tuberculosis suspects at a tertiary care facility in Karachi. In Abstract Book: 45th World Conference on Lung Health of the International Union Against Tuberculosis and Lung Disease; 2014; Barcelona. p. S185.

61. Olarewaju S. Knowledge and practices of community pharmacists on tuberculosis control according to Nigeria guidelines. In Abstract Book: 42nd World Conference on Lung Health of the International Union Against Tuberculosis and Lung Disease; 2011; Lille: Int J Tuberc Lung Dis. p. S355.

62. Carandang RR, Alvarez JP, Aslahon E, Datuemam J, Fernandez R, Mangondato N, et al. Counseling practices on anti-tuberculosis drugs among community pharmacies in Manila. In Abstract Book: 43rd World Conference on Lung Health of the International Union Against Tuberculosis and Lung Disease; 2012; Kuala Lumpur: Int J Tuberc Lung Dis. p. S418.

63. Costello M. Innovative strategies for improving the quality, sustainability, and accessibility of DOTS services in the Philippines. In Abstract Book: 36th World Conference on Lung Health of the International Union Against Tuberculosis and Lung Disease; 2005; Paris: Int J Tuberc Lung Dis. p. S33.

64. Villanueva AE, Costello MP, Mosende ZY, Lardizabal AA, Mangura BT, Reichman LB. Pharmacies as effective allies of the national TB control program. In Abstract Book: 36th World Conference on Lung Health of the International Union Against Tuberculosis and Lung Disease; 2005; Paris: Int J Tuberc Lung Dis. p. S220.

65. Villanueva AE, Mortera LL, Fuentes RM, Palasi WA, De Guzman AM, Mercado RA, et al. Engaging private pharmacies as active partners in TB control: preliminary findings. In Abstract Book: 42nd World Conference on Lung Health of the International Union Against Tuberculosis and Lung Disease; 2011; Lille: Int J Tuberc Lung Dis. p. S327.

66. Mahamba VM, Olotu R, Mkomwa ZH, Nyamkara MF, Egwaga SM. Increasing pediatric tuberculosis detection: moving from guidelines to implementation. In Abstract Book: 44th World Conference on Lung Health of the International Union Aaginst Tuberculosis and Lung Disease; 2013; Paris: Int J Tuberc Lung Dis. p.

S404.

67. Mugyabusu J, Munuo G, Makame M, Kitembe Z, Charlotte C, Helena E. Partnerships with community partnerships in scaling-up TB-HIV services: Kisarawe District experience. In Abstract Book: 42nd World Conference on Lung Health of the International Union Against Tuberculosis and Lung Disease; 2011; Lille: Int J Tuberc Lung Dis. p. S90.

68. Mwangomale AS, Mkomwa ZH, Mahamba V, Mugyabuso JF, Mueller L, Okromeshko S, et al. Tanzania: monitoring and evaluation of community-based efforts to support HIV/TB integration. In Abstract Book: 44th World Conference on Lung Health of the International Union Aaginst Tuberculosis and Lung Disease; 2013; Paris: Int J Tuberc Lung Dis. p. S34.

69. Tarimo A, Kasembe D, Ngowi J, Valimba R, Mwakisu S, Egwaga S, et al. Engaging the retail pharmacies and accredited drug outlets for tuberculosis case detection in Tanzania. In Abstract Book: 44th World Conference on Lung Health of the International Union Aaginst Tuberculosis and Lung Disease; 2013; Paris: Int J Tuberc Lung Dis. p. S37.

70. Jittimanee S, Junmatong A, Nampeng C, Palapol P, Nateniyom S. Waiting too long for spontaneous resolution of cough: health care seeking behaviors among advanced pulmonary tuberculosis patients in urban and rural Thailand. In Abstract Book: 44th World Conference on Lung Health of the International Union Aaginst Tuberculosis and Lung Disease; 2013; Paris: Int J Tuberc Lung Dis. p. S330.

71. Thamsuwan C, Kasetjaroen Y. Comprehensive tuberculosis control through joint effort by the private, civil society and public sectors. In Abstract Book: 45th World Conference on Lung Health of the International Union Against Tuberculosis and Lung Disease; 2014; Barcelona. p. S162-S163.

72. Lonnroth K, Karlsson M, Lan NT, Buu TN, Dieu TT. Referring TB suspects from private pharmacies to the National Tuberculosis Programme: experiences from two districts in Ho Chi Minh City, Vietnam. Int J Tuberc Lung Dis. 2003; 7(12): p. 1147-1153.

73. Lonnroth K, Lambregts K, Nhien DD, Quy HT, Diwan VK. Private pharmacies and tuberculosis control: a survey of case detection skills and reported anti-tuberculosis drug dispensing in private pharmacies in Ho Chi Minh City, Vietnam. Int J Tuberc Lung Dis. 2000; 4(11): p. 1052-1059.

74. Neukom J, Linh CL. Health seeking for tuberculosis symptoms in Dong Tap and An Giang provinces, Viet Nam: the importance of the private sector for increasing case detection. In Abstract Book: 44th World Conference on Lung Health of the International Union Aaginst Tuberculosis and Lung Disease; 2013; Paris: Int J Tuberc Lung Dis. p. S394.

75. Pham HK, Lane-Barlow C, Hennig C. Pricing and availability of anti-tuberculosis drugs in Viet Nam. In Abstract Book: 44th World Conference on Lung Health of the International Union Aaginst Tuberculosis and Lung Disease; 2013; Paris: Int J Tuberc Lung Dis. p. S361.

76. Quy HT, Lan NT, Lonnroth K, Buu TN, Dieu TT, Hai LT. Public-private mix for improved TB control in Hi Chi Minh City, Vietnam: an assessment of its impact on case detection. Int J Tuberc Lung Dis. 2003; 7(5): p. 464-471.

77. Vu DH, van Rein N, Cobelens FG, Nguyen TT, Le VH, Brouwers JR. Suspected tuberculosis case detection and referral in private pharmacies in Viet Nam. Int J Tuberc Lung Dis. 2012; 16(12): p. 1625-1629.
