# Supplementary material for: Engagement of the private pharmaceutical sector for TB control: rhetoric or reality?
Source: J Pharm Policy Pract. 2017 Jan 18;10:6. doi: 10.1186/s40545-016-0093-3 (PMC5241918; doi:10.1186/s40545-016-0093-3)
Supplement: Additional file 3: — Data sources for Fig. 1 – comparison of retail drug outlets with corresponding TB burden and population size. (PDF 113 kb) [file 40545_2016_93_MOESM3_ESM.pdf]

Additional file 3: Data sources for figure 1 - comparison of retail drug outlets with corresponding TB burden and population size

	% of private expenditure on health as Total Health Expenditure*	Incident TB cases**	Incidence per 100,000 people per year**	Estimated number of licensed and unlicensed retail drug outlets	Population#	No. of people per outlet	Drug outlets per 100,000 people
Cambodia	79.5	390	60,000	7,000[a]	15,578,000	2,225	44.94
Afghanistan	78.8	189	60,000	10,131[b]	32,527,000	3,211	31.15
Nigeria	76.1	322	570,000	200,000[c]	182,202,000	911	109.77
Myanmar	72.8	369	200,000	8,000[d]	53,897,000	6,737	14.84
Philippines	68.4	288	290,000	29,000[e]	100,699,000	3,472	28.80
Bangladesh	64.7	227	360,000	200,000[f]	160,996,000	805	124.23
Tanzania	63.7	327	170,000	9,626[g]	53,470,000	5,555	18.00
Pakistan	63.2	270	500,000	65,000[h]	188,925,000	2,907	34.41
Indonesia	61	399	1,000,000	30,643[i]	257,564,000	8,405	11.90
Kenya	58.3	246	110,000	8,200[j]	46,050,000	5,616	17.81
Vietnam	58.1	140	130,000	44,000[k]	91,714,000	2,084	47.98
Uganda	55.6	161	61,000	7,323[l]	39,032,000	5,330	18.76
DR Congo	46.9	325	240,000	3,791[m]	77,267,000	20,382	4.91

* WHO. Global Health Observatory data repository. 2014. http://apps.who.int/gho/data/node.main
** WHO Global TB Report, 2015
# World Bank, 2015

Data sources for estimated number of licensed and un-licensed retail drug outlets

Annex 1 of the 2012 International Pharmaceutical Federation (FIP) Global Pharmacy Workforce Report has data on total number of licensed pharmacies (http://www.fip.org/humanresources) that are based on country surveys. Because we were interested in both licensed and un-licensed retail drug outlets, we expanded our search for data sources because in many low-income and some middle-income countries, retail drug outlets are not adequately licensed or regulated. If data were not available from any other source, we relied on the FIP 2012 Global Pharmacy Workforce Report to determine the estimated number of licensed pharmacies only.

Data sources for estimated number of licensed and unlicensed retail drug outlets

---

[a] Cambodia - The private commercial sector distribution chain for malaria treatment in Cambodia. http://www.actwatch.info/sites/default/files/content/publications/attachments/Cambodia_Report_SCRapid%2520assessment_2009.pdf.

[b] Afghanistan – FIP 2012 Global Pharmacy Workforce Report.

[c] Nigeria - Beyeler N, Liu J, Sieverding M. A systematic review of the role of proprietary and patent medicine vendors in healthcare provision in Nigeria. PLoS One. 2015 Jan 28;10(1):e0117165. doi: 10.1371/journal.pone.0117165.

[d] Myanmar - Lulu.com Myanmar Medical and Pharmaceutical Industry Handbook - Strategic Information and Regulations. Available at https://books.google.com/books?isbn=1312884479.

[e] Philippines - Management Sciences for Health staff based in the Philippines (for estimates of 29,000). A 2013 media article entitled "Public warned on ghost pharmacies" provided lower-end estimates of more than 20,000 drug stores (http://newsinfo.inquirer.net/391941/public-warned-on-ghost-pharmacists).

[f] Bangladesh - Management Sciences for Health staff based in Bangladesh. An upper estimate of 300,000 was also provided, but the team chose the average estimate of 200,000.

[g] Tanzania - Management Sciences for Health staff based in Tanzania. Data include both accredited and non-accredited drug dispensing outlets.

[h] Pakistan - Barriers and Opportunities to Effectively Engage Private Sector Retail Pharmacies as DOTS Centers in Pakistan. http://apps.who.int/medicinedocs/en/m/abstract/Js22356en/

[i] Indonesia - National Health Profile. 2014. http://www.depkes.go.id/resources/download/pusdatin/profil-kesehatan-indonesia/profil-kesehatan-indonesia-2014.pdf.

[j] Kenya - Management Sciences for Health staff based in Kenya.

[k] Vietnam - FIP 2012 Global Pharmacy Workforce Report.

[l] Uganda - Management Sciences for Health staff based in Uganda.

[m] DR Congo - Management Sciences for Health staff based in DR Congo. The estimate of 3,791 retail drug outlets is from only 4 of 11 provinces.
